# Risk Assessment of Microbiological and Chemical Hazards in Foods

**DOI:** 10.3390/foods13131956

**Published:** 2024-06-21

**Authors:** Francesco Esposito, Teresa Cirillo

**Affiliations:** Department of Agricultural Sciences, University of Naples “Federico II”, Via Università, 100, 100-80055 Portici, NA, Italy; tcirillo@unina.it

Food safety constitutes a critical regulatory and quality standard that must be fulfilled by food manufacturers throughout all phases of food production. Ingestion of food presents various risks, with chemical and biological contaminants playing a pivotal role in threatening food hygiene and safety. Therefore, continuous research and improved low-cost detection methods are essential for mitigating these risks and enhancing food safety [1,2]. Main chemical hazards are associated with the presence of endocrine disruptors, heavy metals, and neo-formed chemicals. In contrast, biological hazards stem primarily from consuming food tainted with pathogenic microorganisms. This Special Issue dealt with recent research in the domains of food hygiene and safety, addressing both chemical and biological hazards. It focused particularly on the occurrence of food xenobiotics, including mycotoxins, phthalate esters, process contaminants, and potentially toxic elements, as well as microplastics (MPs), an emerging contaminant halfway between chemical and physical risks, which has drawn significant attention due to its widespread environmental presence and potential health risks. Additionally, biological hazards and innovative detection methods in food products were thoroughly addressed. A central theme of this collection is the contamination of food by potentially toxic elements (PTEs) such as heavy metals, which could be harmful to living organisms and ecosystems, even at low concentrations [3]. Ghidini et al. (contribution 1) investigated the occurrence of PTEs in the muscle and liver of Italian heavy pigs, highlighting the potential health risks associated with dietary exposure among children. The combined consumption of pig liver and muscle could approach the tolerable weekly intake limits for Cd, Fe, and Zn, indicating potential long-term detrimental effects. Likewise, Bacchi et al. (contribution 2) report concerns about toxic metals in East Asian bullfrog legs from Vietnam and Thailand, revealing high levels of As with no significant differences between production areas. indicating potential carcinogenic and non-carcinogenic risks for consumers. This study underscores the role of arsenic-contaminated water as a significant source of As in these organisms. Mycotoxins are a major cause of food losses and represent a recurring food safety challenge [4]. The occurrence of mycotoxins in spices was another critical topic covered in this Special Issue. Nordin et al. (contribution 3) found that spices commonly used in Malaysian cuisine are susceptible to fungal contamination, with coriander seeds (ground) and black pepper (whole) showing the highest levels of fungal presence. Mycotoxigenic fungi such as *A. flavus* and *A. niger* were frequently isolated, indicating a potential risk of mycotoxin exposure for consumers. Further exploring the field of xenobiotics, contaminants from food processing are another crucial theme. Chemical contaminants in food processing can arise from various sources, i.e., high temperatures and certain cooking methods can generate harmful substances such as acrylamide, chloropropanols, and furan [5]. Additionally, contaminants may leach from packaging materials into the food during storage [6], and this aspect has also been investigated in this issue. Pekmezci and Basaran (contribution 4) discuss the implications of heat-treatment contaminants in Turkish diets. This study retrospectively analyzed the 10-year dietary habits of cancer patients, finding significant relationships between dietary heat treatment, contaminant risk scores, and cancer types. Red meat consumption was associated with the highest risk score. Another area of concern is the presence of phthalate esters in coffee. Phthalates are present in various industrial and consumer products, especially plastics. Since they are not chemically bonded to the plastic, they can leach out into the environment, leading to human exposure [7]. Velotto et al. (contribution 5) address the occurrence and risk assessment of phthalate esters in coffee, drawing attention to endocrine disruptors in a widely consumed beverage. This communication deals with the concentration of bis(2-ethylhexyl)phthalate (DEHP) and di-butyl phthalate (DBP) in coffee powder and beverages to ascertain their migration from various packaging and during different brewing methods. While no significant differences were found in phthalate levels among different packaging types, higher DEHP levels were observed in beverages extracted by professional espresso machines compared to Moka pots and home espresso machines.

Turning now to the field of foodborne pathogens, this Special Issue includes three contributions that address the challenges posed by pathogenic bacteria and nematodes. Li et al. (contribution 6) introduce a multiplex PCR system for detecting foodborne pathogens in seafood, offering a promising tool for enhancing food safety. The system described in this study shows effective detection capabilities, confirming its suitability for rapid contamination detection in these foods. Foodborne pathogens and preservation techniques were also examined. Abad et al. (contribution 7) evaluate the efficacy of pulsed electric fields (PEF) in inactivating Anisakis larvae in hake meat. The results indicated that PEF treatment is able to inactivate almost 100% of Anisakis larvae while minimally affecting the quality of hake meat compared to traditional freezing methods, which are commonly employed to mitigate Anisakis-related risk, also taking into account that Anisakis larvae can survive freezing and remain pathogenic, which may explain why some patients develop symptoms after consuming infested frozen fish [8,9]. Ji et al. (contribution 8) evaluated the effects of various organic acids and their combinations on the cell barrier and biofilm of *E. coli*. The findings described in this study highlight the potential for optimizing organic acid combinations for antimicrobial applications in the food industry. This comprehensive Special Issue also includes two intriguing reviews that enhance the contribution to the huge amount of food safety challenges posed by both traditional and emerging contaminants. Microplastics (MPs) in the food chain is a global issue highlighted by Borriello et al. (contribution 9), who examine human exposure to MPs through environmental and dietary sources, underscoring the widespread nature of this challenging concern. This narrative review aims to summarize MPs characteristics, sources, transport pathways, and their ecological and health impacts, identifying human exposure routes. The latter review by Başaran and Çuvalcı (contribution 10) systematically examined sixty-three articles published between January 2002 and April 2022 on the association between dietary acrylamide exposure and cancer risk. While some studies suggested a positive relationship between acrylamide exposure and cancer in various systems and organs, many others found no such link. This paves the way for further research with larger sample sizes and a broader range of foods to provide more reliable results and to advise future health policies. To conclude the interesting collection of articles presented in this Special Issue, an exciting insight into the risk assessment related to the potential consumption of a food industry by-product has also been evaluated, shedding new light on the potentials and challenges of turning waste into a resource. The study by Nolasco et al. (contribution 11) evaluates the safety of coffee silverskin (CS), a by-product of coffee roasting, for its potential use as a food ingredient. The deterministic risk assessment indicated no significant non-carcinogenic or carcinogenic risks related to CS consumption, supporting its potential safe use in functional foods [10].

In summary, this Special Issue not only offers a platform for sharing the latest research in food hygiene and safety but also encourages researchers, policymakers, and industry stakeholders to continue their collaborative efforts in addressing foodborne risks. The variety of topics covered sheds new light on the complexity of ensuring food safety in the modern world and ultimately inspires further investigation and innovation in the pursuit of safeguarding public health and ensuring the integrity of our food systems.

## Data Availability

Not applicable.
